# Persistent Hypoglycemia in Patient with Hodgkin's Disease

**DOI:** 10.1155/2015/820286

**Published:** 2015-12-29

**Authors:** Harold Cinco Lim, Lubna Bashir Munshi, David Sharon

**Affiliations:** ^1^Department of Internal Medicine, Monmouth Medical Center, Long Branch, NJ 07740, USA; ^2^Section of Hematology and Oncology, Monmouth Medical Center, Long Branch, NJ 07740, USA

## Abstract

Hypoglycemia is a rare complication of Hodgkin's disease. Several explanations have been postulated but the exact pathophysiology is not well understood. We are presenting a case of newly diagnosed Stage IV Hodgkin's disease that developed persistent and recurrent hypoglycemia despite giving glucagon, repeated 50% dextrose, and D5 and D10 continuous infusion. Hypoglycemia workup showed the C-peptide level to be low. Patient was suspected of having hypoglycemia related to lymphoma and was given a trial of prednisone which resolved the hypoglycemic episodes and made the patient euglycemic for the rest of his hospital stay. The presence of a substance that mimicked the effects of insulin was highly suspected. Several case reports strengthen the hypothesis of an insulin-like growth factor or antibodies secreted by the cancer cells causing hypoglycemia in Hodgkin's disease but none of them have been confirmed. Further investigation is warranted to more clearly define the pathophysiology of persistent hypoglycemia in patients with Hodgkin's disease.

## 1. Introduction

Hypoglycemia is an uncommon complication of Hodgkin's disease. It has been described in few case reports and is especially more pronounced in cachectic patients. Several explanations have been postulated but the pathophysiology is not well understood. Our case is of a 40-year-old male who had newly diagnosed Stage IV Hodgkin's disease and had recurrent episodes of hypoglycemia during his hospitalization despite continuous intravenous fluid administration with 10% dextrose. Hypoglycemia workup showed the C-peptide level to be low. Patient was suspected of having hypoglycemia related to the lymphoma and was given a trial of prednisone rendering the patient euglycemic. He never had any further episode of hypoglycemia.

## 2. Case Report

A 40-year-old Caucasian male presented with an 8-month history of generalized weakness, fatigue, anorexia, and significant weight loss. Physical examination showed severe cachexia and diffuse nontender posterior cervical, subclavicular, and inguinal lymphadenopathy with a diffuse pruritic maculopapular rash. The rest of the physical examination was within normal limits. On lab work the WBC count was 40100/cu mm, hemoglobin was 5.7 g/dL, hematocrit was 19.6, platelets were 393000/cu mm, MCV was 81.5 fL, segmented polymorphonuclear leucocytes were 98%, glucose was 67 mg/dL, LDH was 947, ESR was 122, CRP was 164.8, and serum electrolytes were within normal limits. Peripheral smear revealed anisocytosis, poikilocytosis, and polychromasia. Reticulocyte count was 3.8%. Iron studies showed ferritin was 1265, iron was 12, and TIBC was 101. Chest X-ray showed right middle lobe and left perihilar infiltrate. CT scan showed extensive lymphadenopathy in the left supraclavicular area, hilum and mediastinum. In the lungs, multiple bilateral masses consistent with metastatic disease were seen. Extensive metastatic disease was seen involving the spleen and liver. Innumerable enlarged lymph nodes were seen throughout the abdomen, pelvis, and inguinal area. Left groin lymph node biopsy revealed large clusters of Reed-Sternberg cells and variants with CD30 and CD15 strongly positive. CD20 was negative. Patient was diagnosed with mixed cellularity Hodgkin's disease. Bone marrow biopsy showed myeloid proliferation but no evidence of Hodgkin's disease. Cytogenetic study was negative for BCR/ABL rearrangement. Echocardiogram in Dec 2013 on initial presentation showed an EF of 25–30%. Given the low EF he was not a candidate for an anthracycline-containing regimen and instead MOPP therapy was initiated. During his hospitalization, he was found to have persistent and recurrent episodes of hypoglycemia requiring repeated doses of intravenous 50% dextrose. Despite multiple doses of dextrose along with good oral intake, the patient had recurrent hypoglycemia with blood sugar levels from low 20 s to low 60 s. He was given glucagon and was started on continuous intravenous fluids with 5% dextrose followed by 10% dextrose. Morning cortisol level was normal and the C-peptide level was low at 0.42 which suggested nonpancreatic insulin source as the cause of hypoglycemia. Patient was started on prednisone and his hypoglycemia resolved and he remained euglycemic for the rest of his hospital stay. The prednisone was tapered slowly. The hypoglycemic episodes never recurred. Upon follow-up at Oncology Clinic and after 4 cycles of MOPP the repeat PET/CT scan showed innumerable partially calcified nodes. He was deemed to have a mixed response to MOPP therapy. As his cardiac function had improved he was placed on standard ABVD chemotherapy with close monitoring of MUGA scans prior to cycle treatment. The patient had another PET/CT scan after the 4 cycles of ABVD showing some response; however a complete resolution was not documented. Due to the suspicion of the remaining active lymphoma the patient underwent a left inguinal node excisional biopsy in Sep 2014 showing sclerotic node with dystrophic calcification and granulomatous reaction. No residual lymphoma was noted. The patient was readmitted in Dec 2014 with intermittent fevers, sweats, and malaise. He had severe anemia requiring transfusions. Marrow biopsy was done showing no marrow involvement. PET/CT scan in Dec 2014 showed persistent widespread lymphadenopathy. He then underwent right axillary lymph node excisional biopsy showing classical Hodgkin's lymphoma-RS cells with bizarre morphology. He then received 2 cycles of ICE salvage chemotherapy. Repeat PET/CT scan after ICE salvage chemotherapy showed marked resolution of the extensive disease. He was eventually sent for stem cell harvesting and underwent autologous stem cell transplant with BEAM conditioning. But all through the follow-up over the year after the discharge since the diagnosis the patient never had any hypoglycemia again. The prednisone resolved the episodes of hypoglycemia all together. The patient till the last follow-up in summer 2015 was still in remission.

## 3. Discussion

There have been several hypotheses proposed for the pathophysiology of hypoglycemia as a complication of Hodgkin's disease but the mechanism has been poorly defined. In a case reported by Pavelić et al. [1], it was noted that, in patients with Hodgkin's disease, hypoglycemia and elevated basal levels of growth hormones in blood were present with elevated levels of Substance Immunologically Cross-Reactive with Insulin (SICRI). Although their observations did not demonstrate that SICRI facilitates glucose uptake by tissues, they speculated that these substances play a role in positive feedback of the endocrine self-control mechanism of tumor growth. The transient tumor associated hypoglycemia stimulates the secretion of growth hormone which further enhances tumor growth stimulating indirectly further uptake of glucose by tumor and other tissues and thus leading to further hypoglycemia. Another case report by Kulkarni et al. [2] speculated that hypoglycemia associated with Hodgkin's disease was probably related to the production of poorly characterized insulin-like substances by malignant cells especially in patients who are cachectic with extensive liver infiltration. Braund et al. [3] reported that a patient with Hodgkin's disease showed impaired in vitro binding of insulin to erythrocyte insulin receptors. The patient developed autoantibodies that inhibited the binding of insulin to the erythrocyte's receptors and stimulated the insulin receptor leading to hypoglycemia. The patient's low blood sugar did not respond to plasmapheresis and azathioprine but it remitted with normalized insulin after administration of prednisone. Walters et al. [4] also reported that the serum of a patient with Hodgkin's disease with severe fasting hypoglycemia has a factor found on the immunoglobulin fraction that enhanced the uptake of glucose in rat adipocytes and these substances displaced the insulin bound in the human erythrocyte and precipitated and phosphorylated human insulin receptors.

Chan et al. [5] postulated that patients with Hodgkin's disease can produce autoantibodies that bind to insulin receptors mimicking insulin effects and hypoglycemia. The hypoglycemia of these patients was abolished after starting on prednisone therapy. Smith et al. [6] presented a case of Hodgkin's disease with persistent hypoglycemia and hyperinsulinemia requiring 10% glucose infusions. Subtotal pancreatectomy with excision of adjacent Hodgkin's disease relieved the hypoglycemia and hyperinsulinemia. Marks et al. and Wanebo et al. [7, 8] described two cases of hypoglycemia associated with Hodgkin's disease. Both patients were cachectic and showed decrease in liver glycogen on autopsy. It was not clear whether hypoglycemia was associated with nonspecific stresses of malnutrition, chemotherapy, tumor effects, or liver disease. Abbasi and Power [9] speculated that the development of hypoglycemia in association with tumors includes excessive glucose uptake by tissue and secretions that impaired hepatic gluconeogenesis and adipose tissue lipolysis. Secretion of insulin or insulin-like factors was thought to be an attractive hypothesis but an extensive review of cases of hypoglycemia related to tumors showed elevated serum insulin and insulin-like activity in only a minority of the cases as stated in the study by Skrabanek and Powell [10]. Another possibility that was postulated was the stimulation of the pancreas by the tumor to secret insulin but an autopsy series by Hart and Hinerman [11] revealed pancreatic islet cell hyperplasia in eight cases of Hodgkin's disease without hypoglycemia. A case by Friedlander [12, 13] demonstrated normal pancreatic architecture with hypoglycemia and postulated that pancreatic secretion was mediated by autonomic nerves in patients with peripancreatic disease.

## 4. Conclusion

In our case we have described a patient who was diagnosed with Hodgkin's disease and who developed recurrent hypoglycemia despite the administration of glucagon, repeated 50% dextrose, and even D5 and D10 continuous infusions. Patient was found to have low C-peptide and when he was started on prednisone, the hypoglycemia remitted and he remained euglycemic from then onwards. The decrease level of C-peptide in our case suggested that the patient did not develop hypoglycemia secondary to insulin secretion by the pancreas, but the presence of a substance that mimicked the effects of insulin was highly suspected. The above cited case reports strengthen the hypothesis of an insulin-like growth factor causing hypoglycemia in Hodgkin's disease but proving this substance to be an autoantibody is not yet confirmed. Further investigation is warranted to more clearly define the pathophysiology of hypoglycemia in patients with Hodgkin's disease.
